# *BUMPY STEM* Is an Arabidopsis Choline/Ethanolamine Kinase Required for Normal Development and Chilling Responses

**DOI:** 10.3389/fpls.2022.851960

**Published:** 2022-04-29

**Authors:** Christina Rabeler, Mingjie Chen, Nick Kaplinsky

**Affiliations:** ^1^Department of Biology, Swarthmore College, Swarthmore, PA, United States; ^2^College of Life Sciences, Xinyang Normal University, Xinyang, China

**Keywords:** choline/ethanolamine kinase, *CEK4*, At2g26830, chilling, pedicel stem junction, phospholipid biosynthesis

## Abstract

Phospholipid biosynthesis is a core metabolic pathway that affects all aspects of plant growth and development. One of the earliest step in this pathway is mediated by choline/ethanolamine kinases (CEKs), enzymes in the Kennedy pathway that catalyze the synthesis of the polar head groups found on the most abundant plant phospholipids. The Arabidopsis genome encodes four *CEK*s. *CEK1-3* have been well characterized using viable mutants while *CEK4* encodes an essential gene, making it difficult to characterize its effects on plant development and responses to the environment. We have isolated an EMS-induced allele of *CEK4* called *bumpy stem* (*bst*). *bst* plants are viable, allowing the effects of decreased *CEK4* function to be characterized throughout the Arabidopsis life cycle. *bst* mutants have a range of developmental defects including ectopic stem growths at the base of their flowers, reduced fertility, and short roots and stems. They are also sensitive to cold temperatures. Supplementation with choline, phosphocholine, ethanolamine, and phosphoethanolamine rescues *bst* root phenotypes, highlighting the flow of metabolites between the choline and ethanolamine branches of the Kennedy pathway. The identification of *bst* and characterization of its phenotypes defines new roles for *CEK4* that go beyond its established biochemical function as an ethanolamine kinase.

## Introduction

Biological membranes compartmentalize cells and, in concert with embedded and associated protein complexes, regulate transport and signal transduction between cells and cellular compartments. Membranes are composed of a phospholipid bilayer consisting of a hydrophobic core sandwiched between hydrophilic head groups. Phosphatidylethanolamine (PtdEtn) and phosphatidylcholine (PtdCho) are the two most abundant phospholipids found in eukaryotic membranes. Their polar head groups, phosphoethanolamine and phosphocholine, are synthesized in the earliest steps of the Kennedy pathway when ethanolamine and choline, respectively, are phosphorylated by choline/ethanolamine kinases (CEKs). Later in the pathway these phosphorylated head groups are enzymatically coupled to the diacylglycerol to create PtdEtn and PtdCho which are subsequently incorporated into membranes (Gibellini and Smith, 2010). Mutations in *CEK*s and other Kennedy pathway enzymes result in a wide range of phenotypes in plants as well as heritable genetic diseases in humans, highlighting the importance of this core biosynthetic pathway in all eukaryotes (Kwon et al., 2012; Lin et al., 2015, 2020; Yunus et al., 2016; Tavasoli et al., 2020; Tannert et al., 2021). Extensive efforts to characterize the Kennedy pathway have revealed that most organisms have multiple CEK isoforms encoded by distinct *CEK* genes.

The Arabidopsis genome encodes four *CEK*s numbered *CEK1-4*. These genes are expressed at different levels compared to each other and exhibit tissue-specific and environmentally responsive expression patterns, suggesting that each enzyme plays a distinct role in choline and ethanolamine metabolism (Tasseva et al., 2004; Lin et al., 2020). Examples of sub-specialization in this gene family include diverse substrate specificities among the CEKs as well as their differential effects on plant growth and development. *In vitro*, *CEK1* and *CEK2* have both choline and ethanolamine kinase activity while *CEK3* and *CEK4* have substrate specificity for choline and ethanolamine alone, respectively. Neither *CEK1* or *CEK2* exhibited detectable ethanolamine kinase activity *in vivo*, suggesting that *CEK4* is the principal ethanolamine kinase in Arabidopsis (Lin et al., 2019, 2020). Similar enzymatic specialization has also been described in *Glycine max*, suggesting that it is widespread in plants (Wharfe and Harwood, 1979). Complementing this biochemical approach, reverse genetics has also been used to reveal the developmental and biochemical roles of the Arabidopsis CEKs. *cek2* and *cek3* single mutants have short roots while *cek1* mutant roots are normal. This shows that at least two of the Arabidopsis *CEK*s are required for normal growth and development. Additional insights into CEK function have come from a chemical rescue experiment. In this experiment *cek3* seedlings were grown on media containing phosphocholine, which rescued the *cek3* short-root phenotype. This experiment’s results are consistent with the biochemical characterization of *CEK3* as a choline kinase. *cek* mutants have also been used to provide *in vivo* support for the *in vitro* biochemical characterization. *cek1* single mutant and *cek1 cek3* double mutant plants were shown to have decreased choline kinase activity *in vivo* (Lin et al., 2020).

Compared to *CEK1-3*, *CEK4* remains relatively uncharacterized. This is because *cek4* loss of-function mutants are embryo lethal (Meinke et al., 2008; Lin et al., 2015; Meinke, 2020). The viability of *cek1*, *cek2*, and *cek3* T-DNA loss-of-function mutants coupled with the lack of detectable changes in lipid content in their single mutants suggests that *cek1-3* have partially overlapping functions in lipid metabolism and plant development (Lin et al., 2015, 2020). *CEK4* stands out from the other Arabidopsis *CEK*s as the functional alleles of *CEK1*, *CEK2*, and *CEK3* in *cek4* mutants cannot compensate for the loss of *CEK4* activity. Fundamental differences between *CEK4* and the other *CEKs* are also suggested by the observation that *CEK4* is phylogenetically distant from the other Arabidopsis *CEK*s (Lin et al., 2015). Combined with *CEK4*’s ethanolamine substrate specificity *in vitro*, it appears that *CEK4* plays unique and important roles in plant biochemistry and development. These roles are only partially understood. *CEK4* is expressed throughout the plant, including throughout heart stage and later embryos. High expression levels are found in the reproductive organs and in pollen, roots, and trichomes (Honys and Twell, 2004; Marks et al., 2009; Gilding and Marks, 2010; Lin et al., 2015). A 35S:*CEK4* line that complemented *cek4* embryonic arrest was shown to result in increased levels of both PtdEtn and PtdCho in seedlings and increased levels of PtdCho in mature siliques, consistent with a predicted function as a CEK (Lin et al., 2015). No further phenotypes were reported for this overexpression line and, to the best of our knowledge, no roles in post-embryonic plant development or responses to the environment have been described for *CEK4*.

Here we describe the isolation of a viable EMS induced allele of *CEK4* that appears to result in a partial loss of function. This novel allele reveals important post-embryonic developmental functions as well as a role in chilling responses for *CEK4*.

## Materials and Methods

### EMS Mutagenesis

EMS mutagenesis was performed as described in Silverblatt-Buser et al. (2018).

### Plant Growth

Plants were grown on soil under standard long-day greenhouse conditions with supplemental lighting. Plants grown on plates were grown on 0.5× Murashige and Skoog (0.5× MS) media containing 1% sucrose at 22°C under constant light conditions in E-30B growth chambers (Percival Scientific).

### *bumpy stem* Mapping and Cloning

DNA from 412 individuals with mutant phenotypes in a *bst* mapping population was prepared using a DNeasy Plant Maxi Kit (Qiagen). A NEBNext DNA library prep set for Illumina (NEB) was used to prepare the sequencing library that was sequenced using an Illumina HiSeq (Illumina). Reads were mapped to the TAIR10 reference genome using SHORE and *bst* was mapped using SHOREmap (Schneeberger et al., 2009).

### Scanning Electron Microscopy

Fresh tissue was imaged using low vacuum mode on a Quanta 200 scanning electron microscope (FEI) equipped with a cooled stage.

### Chemical Complementation

Ler and *bst* seeds were sterilized and plated on 0.5× MS media, stratified at 4°C for 2 days, and transferred to an incubator for vertical growth at 22°C. After 3 days, seedlings were transferred to treatment plates containing either 100 μM or 1 mM of choline chloride (Cho), phosphocholine chloride calcium salt tetrahydride (PCho), monoethanolamine (MEA), or *O*-phosphorylethanolamine (PEA) (Sigma, St. Louis, MO, United States). The position of root tips were marked at transplanting. Seven days after transplanting the plates were scanned using an Epson V600 scanner with a black piece of felt as a background and root growth was measured using ImageJ (Schneider et al., 2012). These experiments were repeated three times with 10–12 seedlings in each replicate.

### Rosette Size Measurements

Rosette sizes were measured from images using ImageJ (Schneider et al., 2012) by measuring the Feret diameter of the smallest oval that completely surrounded each rosette.

## Results

### *bumpy stem* Is a Recessive Mutant With Pleiotropic Developmental Phenotypes

*bumpy stem* (*bst*) was identified as a mutant with bumpy and twisted stem during a genetic screen unrelated to phospholipid head group biosynthesis. In this screen, *bob1-3* mutants in a Ler background were mutagenized with EMS and screened for synthetic developmental phenotypes (Silverblatt-Buser et al., 2018). Although *bst* does not exhibit a genetic interaction with *bob1-3*, we decided to characterize and clone the mutant gene because it had such an unusual phenotype. We back crossed *bst* into both Col-0 and Ler five times in order to remove *bob1-3* and other unlinked EMS induced mutations and their phenotypes from our analysis. *bst* phenotypes, while similar in both Col-0 and Ler backgrounds, are consistently stronger in Ler. F_1_ plants of *bst*/*bst* × +/+ crosses did not display *bst* phenotypes and *bst* phenotypes segregate in a mendelian fashion in F_2_ families. This suggests that they are caused by a single recessive mutation. The most striking phenotype in *bst* mutants is the abnormal patterning of the inflorescence stem.

**FIGURE 1:**
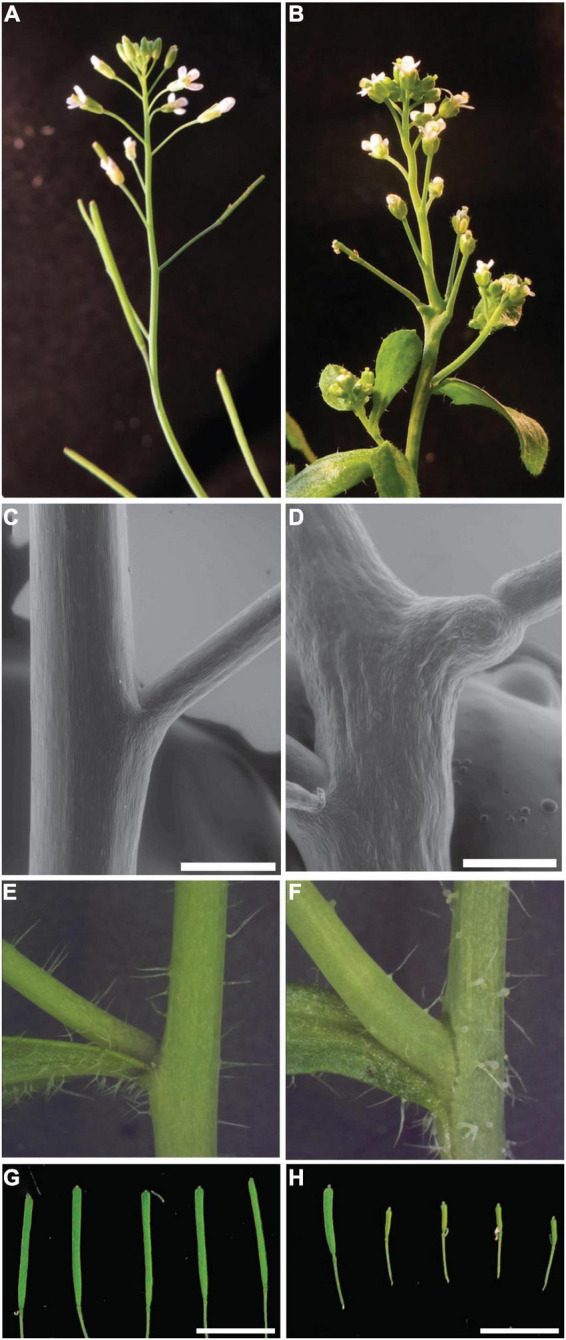
*bumpy stem* inflorescence phenotypes. Normal Col-0 inflorescences exhibit spiral phyllotaxy with flowers separated from each other along the inflorescence **(A)**. *bst* flowers are found in clusters on the inflorescence and exhibit disrupted phyllotactic patterning **(B)**. The boundary between the pedicel and stem is flush with the side of the stem in Col-0 plants **(C)**. In *bst* mutants the notch defining the boundary is displaced, occurring at the tip of ectopic stem tissue that extends from the side of the stem **(D)**. No displacement is observed at the boundary between the main stem and axillary stems **(E,F)**. Compared to Col-0 trichomes **(E)**, *bst* trichomes have swollen bases **(F)**. Wild-type Ler plants are fertile and have filled and expanded siliques **(G)**. *bst* mutants in a Ler background are semi sterile, resulting in short siliques containing few seeds (left silique) and many sterile unexpanded siliques (right siliques) **(H)**. Scale bars are 0.5 mm in **C,D** and 1 mm in **G,H**.

*bumpy stem* inflorescence stems are irregularly swollen and bumpy compared to Col-0 inflorescence stems. In Col-0 plants the phyllotactic patterning of flowers on the flanks of the stem is regular (Figure 1A). The regular spacing between flowers observed in Col-0 plants is irregular in the mutant, resulting in clusters of flowers with disrupted phyllotactic patterning (Figure 1B). Closer inspection of the stem/pedicel boundary revealed a disruption of this boundary in *bst* plants. Col-0 plants have a distinct boundary between the stem and the floral pedicels. This boundary lines up with the lateral edge of the stem (Figure 1C). In *bst* plants this boundary is moved away from the edge of the stem. Unlike wild-type plants, *bst* plants have bumps of tissue that extend from the edge of the stem. The boundary between this ectopic tissue and the floral pedicels is a clearly visible notch separating the stem bumps and the pedicels. Instead of being in line with the edge of the stem, this boundary is displaced laterally (Figure 1D). In addition to flowers, Arabidopsis inflorescences also have secondary or axillary inflorescences that emerge from their flanks. The boundary between axillary inflorescences and the primary inflorescence stem is similar in *bst* and Col-0 plants with no ectopic growth subtending *bst* axillary inflorescences (Figures 1E,F).

*bumpy stem* plants have several other phenotypes associated with their inflorescences. Compared to the trichomes found on Col-0 stems *bst* trichomes have swollen bases (Figures 1E,F). *bst* plants are also semi-sterile. In the more severe Ler background only some siliques (16%, *n* = 93), most frequently from later flowers, produce seeds. The *bst* siliques that do make seeds and elongate are significantly shorter than Ler siliques (Ler 1.23 ± 0.15 cm, *bst* 0.82 ± 0.19 cm, two-tailed *t*-test *p* < 1 × 10^–5^, Figures 1G,H). In the less severe and more fertile Col-0 background all siliques contain seeds. However, *bst* siliques are shorter than wild-type siliques (Col-0 1.36 ± 0.18 cm, *bst* 0.57 ± 0.24 cm, two-tailed *t*-test *p* < 1 × 10^–4^). In addition to these phenotypes, *bst* mutants also have smaller leaves, shorter inflorescences, and shorter roots compared to normal plants (Figures 3, 4).

**FIGURE 2:**
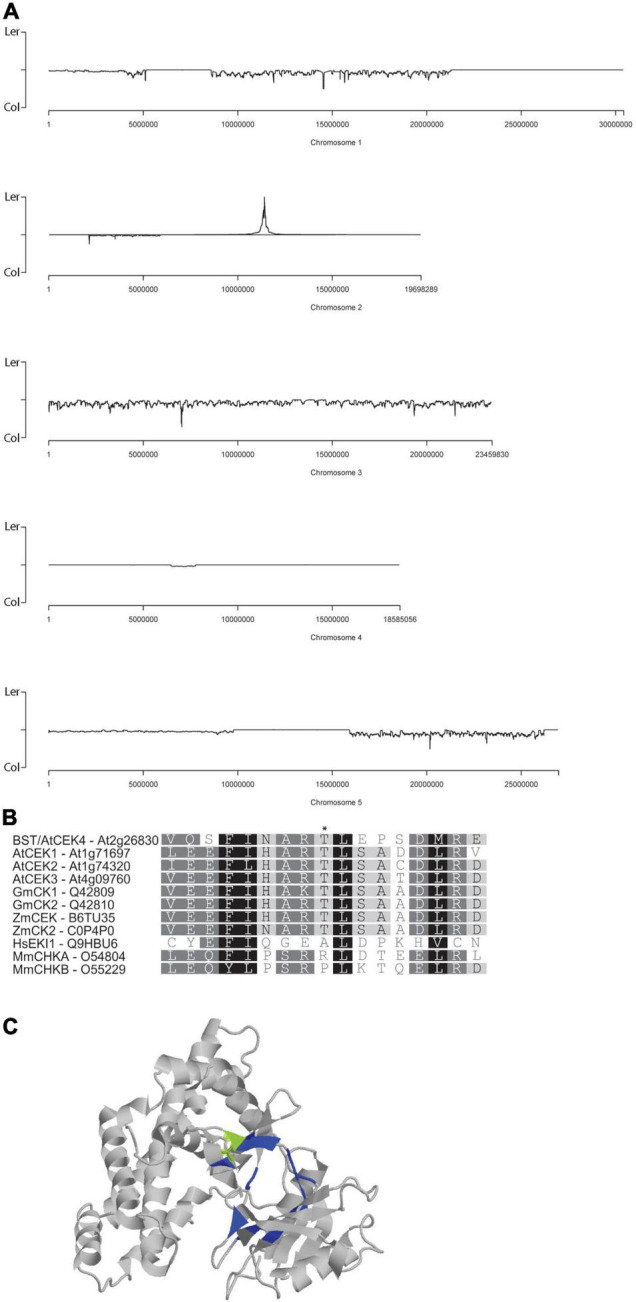
Cloning and characterization of *bst*. *bst* was simultaneously mapped and cloned using a NGS approach. *bst* was generated in a Ler background and its position on chromosome 2 can be seen as peak of enrichment in Ler SNPs **(A)**. A protein alignment of CEKs from Arabidopsis (At), soybean (Gm), maize (Zm), humans (Hs), and mice (Mm) centered on T144 (indicated with a *) which is the site of the T144I mutation in *bst* **(B)**. CEK4 structure with the ATP binding pocket visible. Residues that participate in ATP binding are shaded blue and T141 is shaded green. Image generated using the molecule viewer at bar.utoronto.ca/eplant (Waese et al., 2017) **(C)**.

**FIGURE 3:**
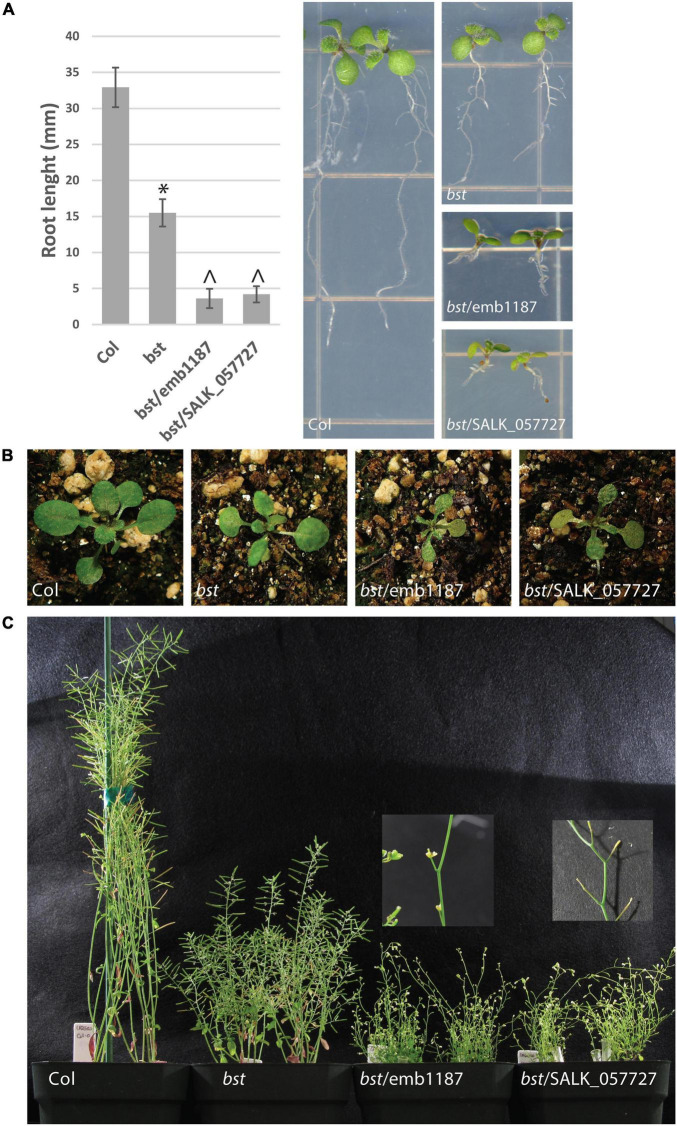
*bumpy stem* root and shoot phenotypes and genetic complementation. *bst* plants were crossed to heterozygous emb1187 and SALK_057727 plants, both of which contain T-DNA insertions in *CEK4*. Seven day old *bst*/*bst* plants were imaged and their root lengths were measured. Root lengths were compared using unpaired two-tailed *t*-tests. The symbol “*” indicates a difference from Col with a *p*-value < 10^– 10^. The symbol “^” indicates a difference from both Col and *bst* with a *p*-value < 10^– 7^. Error bars are SD **(A)**. *bst* vegetative rosettes are smaller than wild-type. This phenotype is enhanced when the *bst* allele is combined with either *emb1187* or SALK_057727 **(B)**. The height of mature plants is reduced in *bst* plants and even more so in *bst*/*emb1187* and *bst*/SALK_057727 plants. *bst*/*emb1187* and *bst*/SALK_057727 plants are sterile **(C)**.

**FIGURE 4:**
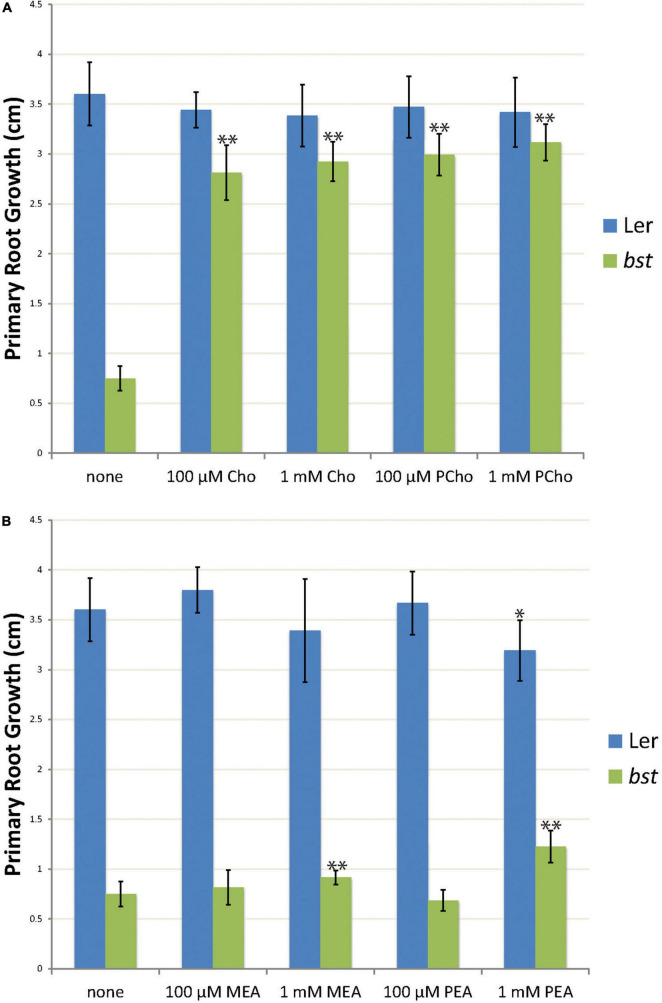
The effects of choline, phosphocholine, ethanolamine, and phosphoethanolamine supplementation on root growth. Three day old seedlings were transplanted to plates containing choline (Cho) or phosphocholine (PCho) **(A)** or ethanolamine (MEA) or phosphoethanolamine (PEA) **(B)**. Primary root growth was measured after seven days of growth. The root length of treated plants was compared to untreated plants of the same genotype using unpaired *t*-tests. “*” indicates a *p*-value < 0.01 and “**” indicates a *p*-value < 0.001. Error bars are SD.

### A Point Mutation in the Choline/Ethanolamine Kinase At2g26830/*CEK4*/*emb1187* Causes *bumpy stem* Phenotypes

To map and clone *bst* we generated a mapping population by crossing *bst* twice into a Col-0 background. Pooled genomic DNA from 412 mutants isolated from this mapping population was sequenced to ∼100-fold coverage. SHOREmap (Schneeberger et al., 2009) was used to simultaneously map and identify putative causal mutations in the sequence data using Col-0/Ler SNPs as mapping markers. A single mapping peak was identified at 11.4 Mb on chromosome 2 (Figure 2A). Of the two non-synonymous mutations within 250 kb of the mapping peak, the 422C>T mutation in At2g26830 turned out to be the *bst* causal mutation (see below). The mutation results in a T141I substitution, converting the polar threonine residue at this position into isoleucine (which is hydrophobic). The threonine at position 141 in *CEK4* is invariant in the four Arabidopsis *CEK*s as well as in corn (*Zea mays*) and soybean (*G. max*) *CEK*s (Figure 2B). It lies outside of the CEK catalytic domains d-6 and d-7 (Aoyama et al., 2004) at a position that directly interacts with ATP and forms part of the nucleotide binding pocket in the crystalized human choline kinase (Malito et al., 2006; Figure 2C).

In order to confirm that the mutation in *CEK4* was responsible for *bst* phenotypes we used two previously identified *cek4* T-DNA alleles in a genetic complementation experiment. Both alleles have embryo-lethal phenotypes. The first, *cek4-1* (SALK_057727), arrests at the heart stage (Lin et al., 2015). The second, *emb1187* (CS16107), arrests at the globular stage (Meinke et al., 2008; Meinke, 2020). As both of these T-DNA alleles are in a Col-0 background, we crossed each to *bst* introgressed into Col-0. Both lines failed to complement *bst* mutant phenotypes including short roots, small rosettes, short inflorescences, and bumpy stems. Each of these phenotypes was more severe in *bst*/*emb1187* and *bst*/*SALK_057727* plants than in *bst*/*bst* plants and the transheterozygous plants were completely sterile (Figure 3). The enhancement of the *bst* phenotype by two loss of function T-DNA alleles demonstrates that genetically, *bst* is a partial loss of function or hypomorphic allele (Muller, 1932).

### Supplementation With Choline, Phosphocholine, Ethanolamine, and Phosphoethanolamine Rescues *bumpy stem* Root Growth Phenotypes

CEK4 has ethanolamine kinase activity *in vivo* and does not appear to function as a choline kinase (Lin et al., 2020). We took advantage of the viability of *bst* plants to test the hypothesis that *CEK4* functions as an ethanolamine kinase in plants using a chemical supplementation approach. The simplest model is that *bst* phenotypes are caused by a lack of ethanolamine kinase activity. This would make low phosphoethanolamine levels the proximal deficiency causing phenotypes. If this is the case then phosphoethanolamine supplementation should rescue *bst* phenotypes, just as phosphocholine can rescue *cek3* root phenotypes and ethanolamine and choline can rescue *serine decarboxylase 1* mutants (Kwon et al., 2012; Lin et al., 2020). We decided to test this by quantitating root growth rates in wild-type and *bst* seedlings after supplementing them with polar head group precursors. Seedlings in a Ler background were germinated and then transferred to plates containing either choline, phosphocholine, ethanolamine, or phosphoethanolamine. Treatment with 100 μM choline or phosphocholine resulted in significant increases in *bst* root growth rates, nearly completely rescuing the short root mutant phenotype. The same effect was observed at 1 mM concentrations of choline and phosphocholine, suggesting that 100 μM concentrations are sufficient for the observed phenotypic rescue (Figure 4A). In contrast, treatment with 100 μM phosphoethanolamine or ethanolamine did not significantly change *bst* root growth rates. Increasing the concentration of ethanolamine or phosphoethanolamine to 1 mM resulted in significant increases in *bst* root growth rates. However, the effect of ethanolamine or phosphoethanolamine supplementation at 1 mM was much smaller than that seen with either choline or phosphocholine treatment at either 100 μM or 1 mM (Figure 4B).

### *bumpy stem* Mutants Have a Chilling Sensitive Phenotype

**FIGURE 5:**
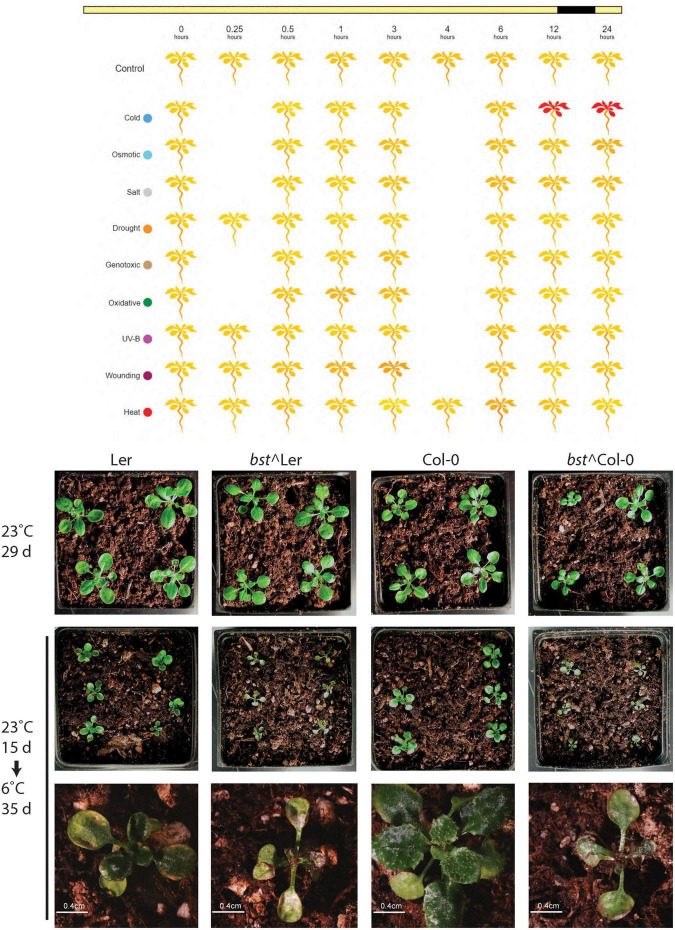
*bumpy stem* responses to low temperatures. *bst* expression is induced by cold temperatures but not by other abiotic stresses. Yellow indicates low expression and red indicates high expression [data from Kilian et al. (2007), image generated using eplant at bar.utoronto.ca/eplant (Waese et al., 2017)]. Normal plants and *bst* plants in both Ler or Col-0 backgrounds were grown for 15 days at 23°C. Untreated plants were grown for an additional 14 days at 23°C while cold treated plants were transferred to 6°C and grown for an additional 35 days.

The composition of phospholipid bilayers is known to change as a compensatory response to a wide range of abiotic stresses. Temperature extremes, both high and low, result in alterations in membrane lipids (Welti et al., 2002). Gene expression profiling in response to a range of abiotic stresses showed that *CEK4* is strongly induced by cold temperatures (4°C) but not by other abiotic stresses (Figure 5; Kilian et al., 2007). This suggests that *CEK4* may have important functions in low temperature responses and that *bst* mutants might have chilling inducible phenotypes. To test this hypothesis we germinated and grew plants on soil for 15 days at 23°C before transferring them to 6°C for an additional 35 days. *bst* plants in both Col-0 and Ler backgrounds grew similarly to wild-type when grown at 23°C for 29 days. Differences between wild-type and *bst* plants were apparent after 35 days of growth at 6°C. After extended growth at low temperatures, *bst* plants were stunted compared to wild-type plants and their leaves were covered with cold induced lesions (Figure 5). While every Col-0 and Ler plant grown at 6°C continued to make new leaves that expanded and turned green, none of the *bst* plants in either genetic background did so. Instead, their apices became discolored and ceased to produce visible new leaves. The rosette sizes of cold grown plants were significantly smaller than those of untreated plants for all genotypes and growth reductions were larger in *bst* than in normal plants in both genetic backgrounds (Ler 55% reduction, *bst*^Ler 63% reduction, Col-0 46% reduction, *bst*^Col-0 55% reduction, two-tailed *t*-test *p* < 0.002 for all genotypes). The interaction between genotype and treatment on rosette size was significant in a Ler background [*f*(1) = 6.414, *p* = 0.015] but not in a Col background, consistent with the increased severity of other phenotypes in Ler.

## Discussion

*CEK4* is the least well characterized choline/ethanolamine kinase in Arabidopsis because it is required for embryonic viability and, up to now, only T-DNA loss of function alleles have been available. This has made it hard to establish what roles it plays in plant growth and development. In this study we isolated *bst*, a partial loss of function allele of *CEK4*, and used it to demonstrate that *CEK4* plays roles in post-embryonic developmental patterning and cold responses.

*CEK4* had previously been shown to be required for embryogenesis as *cek4* T-DNA mutant embryos arrest early during development (Lin et al., 2015). While it is not surprising that a key enzyme in a core biosynthetic pathway would be an essential gene, there are three other *CEK* genes in the Arabidopsis genome which could compensate for a loss of CEK4 activity. The observation that they don’t suggests that *CEK4* has specialized functions that overlap minimally, if at all, with those of the other *CEK*s. The *bst* allele of *CEK4* behaves as a partial loss of function, demonstrated by the increased phenotypic severity when it is combined with a loss of function allele. *bst* is a point mutant, altering a key residue in the ATP binding pocket of the enzyme. The biochemical consequences of this change are uncharacterized but presumably the mutation results in decreased kinase activity due to altered ATP binding. This hypothesis could be tested in the future by directly assaying the effects of the *bst* T141I substitution on ATP binding and ethanolamine kinase activity, either *in vitro* or *in vivo*. We have not determined if *bst* mutant plants have alterations in their membranes. An analysis of any alterations may provide insights into the role that *CEK4* plays in the biological pathways that are affected in *bst* mutants.

*bumpy stem* plants have pleiotropic phenotypes, consistent with *CEK4* expression throughout plant development. These phenotypes include short roots and stems, swollen trichome bases, partial sterility, stem bumps at stem/pedicel junctions, and decreased chilling tolerance. These results suggest that the correct flux of metabolites through the Kennedy pathway is important not only for membrane biosynthesis but also for a variety of higher order processes in plants. Although we did not generate mechanistic insights into how *bst* results in decreased chilling tolerance, there are examples that illustrate the role of temperature dependent changes in phospholipid composition on temperature dependent responses. One example is temperature dependent flowering. The florigen *FT* has been shown to bind to membrane phospholipids at low temperatures, preventing it from moving and functioning as a signal. This serves as a mechanism that allows plants to delay flowering in response to chilling (Nakamura et al., 2014; Susila et al., 2021). Changes in the phospholipid composition of membranes could have direct functional effects on the protein machinery embedded in or associated with them which, in turn, may alter intra- and inter-cellular signaling (Champeyroux et al., 2020). In addition, there are many well established examples of lipids functioning directly as signaling molecules in plants (Wang and Chapman, 2013; Ruelland and Valentova, 2016). These examples include roles for lipids in responding to abiotic stresses such as chilling and freezing responses (Welti et al., 2002; Hou et al., 2016).

**FIGURE 6:**
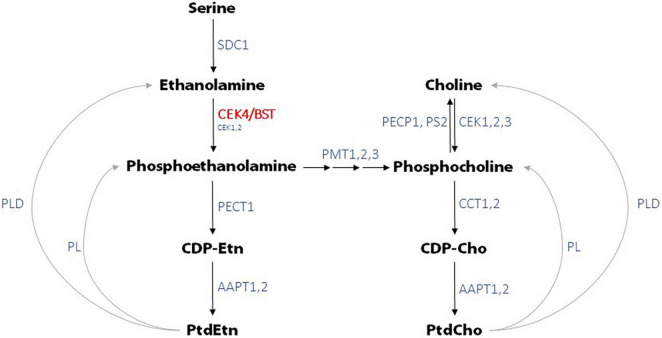
Phosphatidylethanolamine (PtdEtn) and phosphatidylcholine (PtdCho) biosynthetic pathways in Arabidopsis. CEK4/BST functions as the principal ethanolamine kinase, directly producing phosphoethanolamine which is used to generate PtdEth. Through the activity of PMTs, CEK4/BST is also responsible for the production of PtdCho and other choline containing compounds. Enzymes not defined in the text are SDC1, serine decarboxylase 1; PECT1, CTP:phosphorylethanolamine cytidylyltransferase 1; AAPT1,2, aminoalcohol aminophosphotransferase 1,2; PL, phospholipase; PLD, phospholipase D; PECP1, phosphoethanolamine/phosphocholine phosphatase 1; PS2, phosphate starvation-induced gene 2; CCT1,2, CTP:phosphorylcholine cytidylyltransferase 1,2. CDP-Cho is cytidine diphosphocholine and CDP-Etn is cytidine diphosphoethanolamine. Adapted from models presented in Lin et al. (2015), Chen et al. (2019), and Liu et al. (2019).

CEK4 has been shown to have ethanolamine kinase but no detectable choline kinase activity *in vitro* (Lin et al., 2020). This does not mean that mutations in *CEK4* will only affect phosphoethanolamine production as phosphoethanolamine can be converted to phosphocholine by phosphoethanolamine *N*-methyltransferases (PMTs) (Figure 6). The observation that *pmt1* mutants have short roots (Cruz-Ramírez et al., 2004) demonstrates that normal levels of phosphocholine, produced from phosphoethanolamine, are required for robust root growth. Arabidopsis *pmt1 pmt3* double mutant plants have reduced phosphocholine levels and exhibit developmental phenotypes including small roots and shoots, but not bumpy stems, that can be rescued by choline or phosphocholine supplementation (Chen et al., 2018, 2019). *pmt1 pmt2 pmt3* triple mutants are seedling lethal and exhibit up to 80% reductions in phosphocholine levels. This suggests that the bulk, if not all of, the phosphocholine found in Arabidopsis is produced from phosphoethanolamine and that decreased ethanolamine kinase activity will result in decreased phosphocholine levels (Liu et al., 2018, 2019; Chen et al., 2019). The conversion of phosphoethanolamine to phosphocholine by PMTs probably explains why *CEK4* overexpression in Arabidopsis resulted in both increased PtdEth as well as increased PtdCho levels even though *CEK4* only has ethanolamine kinase activity (Lin et al., 2015).

We tested the prediction that the *bst* short root phenotype would be rescued by supplementation with phosphoethanolamine, CEK4’s predicted product. Surprisingly, phosphoethanolamine only partially rescued *bst* root phenotypes and only did so at relatively high concentrations (1 mM). The same was true for ethanolamine, CEK4’s predicted substrate. We also supplemented *bst* seedlings with choline and phosphocholine. Rescue with both of these compounds was nearly complete at relatively low concentrations (100 μM). These unexpected results do not provide clear insights into *bst*’s root phenotypes; understanding them better will require a detailed metabolic analysis. One possible interpretation of these results is that, although *CEK4* functions principally or solely as an ethanolamine kinase, much of the phosphoethanolamine it produces is subsequently used as a substrate for the choline branch of the Kennedy pathway (Figure 6). Since a reduction in PMT generated phosphocholine results in short roots (Cruz-Ramírez et al., 2004; Chen et al., 2019; Liu et al., 2019), the *bst* phenotype could be principally due to reduced levels of choline containing compounds as opposed to ethanolamine containing compounds. This could explain the rescue with lower concentrations of choline containing compounds than ethanolamine containing compounds. Presumably only some of the supplemental phosphoethanolamine would be converted to phosphocholine by PMTs, reducing the efficiency of rescue with ethanolamine containing compounds relative to choline containing compounds. This idea could be tested by supplementation with labeled compounds followed by a metabolic analysis. Combining such an analysis with chemical inhibition of PMTs (Liu et al., 2019) or by using *bst pmt* double mutants could further refine our understanding of the flux of metabolites in the Kennedy pathway on plant growth and development. When combined with mutations in other *CEK* genes, *bst* may also provide insights into the *in planta* functions of this important gene that are shared with the other *CEK*s.

The viability of the *bst* allele provides a useful tool for further investigation of *CEK4* functions in development and responses to the environment. This study did not attempt to define the defects that underlie the developmental, reproductive, or chilling phenotypes we identified. Of special interest is the bumpy stem phenotype that this allele was named for. This phenotype is unusual and we are not aware of other Arabidopsis mutants that display this phenotype. Organ boundaries in plants are delineated by *LOB* gene expression (Shuai et al., 2002) and regulated by a complex genetic network that includes *CUC* and other genes (Hepworth and Pautot, 2015). Investigating the expression domains of *LOB*, *CUC*, and other boundary network genes in *bst* mutants could provide insights into the developmental signaling mechanisms that link phospholipid head group metabolism and the establishment of boundaries between organs.

## Data Availability Statement

Strains described in this manuscript have been deposited at the ABRC with accession numbers CS73151 (*bst*^Col-0) and CS73152 (*bst*^Ler). The sequence data generated in the course of mapping and cloning *bst* have been deposited in the NCBI SRA as BioProject PRJNA796015.

## Author Contributions

CR and MC conceived of and performed the experiments and edited the manuscript. NK conceived of and performed the experiments, supervised the work, wrote the original draft, and edited the manuscript. All authors contributed to the article and approved the submitted version.

## Conflict of Interest

The authors declare that the research was conducted in the absence of any commercial or financial relationships that could be construed as a potential conflict of interest.

## Publisher’s Note

All claims expressed in this article are solely those of the authors and do not necessarily represent those of their affiliated organizations, or those of the publisher, the editors and the reviewers. Any product that may be evaluated in this article, or claim that may be made by its manufacturer, is not guaranteed or endorsed by the publisher.

## References

[B1] Aoyama C. Liao H. Ishidate K. (2004). Structure and function of choline kinase isoforms in mammalian cells. *Prog. Lipid Res.* 43 266–281. 10.1016/j.plipres.2003.12.001 15003397

[B2] Champeyroux C. Stoof C. Rodriguez-Villalon A. (2020). Signaling phospholipids in plant development: small couriers determining cell fate. *Curr. Opin. Plant Biol.* 57 61–71. 10.1016/J.PBI.2020.05.007 32771964

[B3] Chen W. Salari H. Taylor M. C. Jost R. Berkowitz O. Barrow R. (2018). NMT1 and NMT3 N-Methyltransferase Activity Is Critical to Lipid Homeostasis, Morphogenesis, and Reproduction. *Plant Physiol.* 177 1605–1628. 10.1104/PP.18.00457 29777000 PMC6084668

[B4] Chen W. Taylor M. C. Barrow R. A. Croyal M. Masle J. (2019). Loss of phosphoethanolamine n-methyltransferases abolishes phosphatidylcholine synthesis and is lethal. *Plant Physiol.* 179 124–142. 10.1104/pp.18.00694 30381317 PMC6324220

[B5] Cruz-Ramírez A. López-Bucio J. Ramírez-Pimentel G. Zurita-Silva A. Sánchez-Calderon L. Ramírez-Chávez E. (2004). The xipotl Mutant of Arabidopsis Reveals a Critical Role for Phospholipid Metabolism in Root System Development and Epidermal Cell Integrity. *Plant Cell* 16 2020–2034. 10.1105/TPC.103.018648 15295103 PMC519195

[B6] Gibellini F. Smith T. K. (2010). The Kennedy pathway-De novo synthesis of phosphatidylethanolamine and phosphatidylcholine. *IUBMB Life* 62 414–428. 10.1002/iub.337 20503434

[B7] Gilding E. K. Marks M. D. (2010). Analysis of purified glabra3-shapeshifter trichomes reveals a role for NOECK in regulating early trichome morphogenic events. *Plant J.* 64 304–317. 10.1111/J.1365-313X.2010.04329.X 21070410

[B8] Hepworth S. R. Pautot V. A. (2015). Beyond the divide: boundaries for patterning and stem cell regulation in plants. *Front. Plant Sci.* 6:1052. 10.3389/fpls.2015.01052 26697027 PMC4673312

[B9] Honys D. Twell D. (2004). Transcriptome analysis of haploid male gametophyte development in Arabidopsis. *Genome Biol.* 5 1–13. 10.1186/GB-2004-5-11-R85 15535861 PMC545776

[B10] Hou Q. Ufer G. Bartels D. (2016). Lipid signalling in plant responses to abiotic stress. *Plant Cell Environ.* 39 1029–1048. 10.1111/PCE.12666 26510494

[B11] Kilian J. Whitehead D. Horak J. Wanke D. Weinl S. Batistic O. (2007). The AtGenExpress global stress expression data set: protocols, evaluation and model data analysis of UV-B light, drought and cold stress responses. *Plant J.* 50 347–363. 10.1111/j.1365-313X.2007.03052.x 17376166

[B12] Kwon Y. Yu S. Lee H. Yim J. H. Zhu J.-K. K. Lee B. H. (2012). Arabidopsis Serine Decarboxylase Mutants Implicate the Roles of Ethanolamine in Plant Growth and Development. *Int. J. Mol. Sci.* 13 3176–3188. 10.3390/ijms13033176 22489147 PMC3317708

[B13] Lin Y. C. Araguirang G. E. Ngo A. H. Lin K. T. Angkawijaya A. E. Nakamura Y. (2020). The four Arabidopsis choline/ethanolamine kinase isozymes play distinct roles in metabolism and development. *Plant Physiol.* 183 152–166. 10.1104/pp.19.01399 32205454 PMC7210642

[B14] Lin Y.-C. Kanehara K. Nakamura Y. (2019). Arabidopsis CHOLINE/ETHANOLAMINE KINASE 1 (CEK1) is a primary choline kinase localized at the endoplasmic reticulum (ER) and involved in ER stress tolerance. *New Phytol.* 223 1904–1917. 10.1111/nph.15915 31087404

[B15] Lin Y. C. Liu Y. C. Nakamura Y. (2015). The choline/ethanolamine kinase family in arabidopsis: essential role of CEK4 in phospholipid biosynthesis and embryo development. *Plant Cell* 27 1497–1511. 10.1105/tpc.15.00207 25966764 PMC4456650

[B16] Liu Y. C. Lin Y. C. Kanehara K. Nakamura Y. (2018). A pair of phospho-base methyltransferases important for phosphatidylcholine biosynthesis in Arabidopsis. *Plant J.* 96 1064–1075. 10.1111/TPJ.14090 30218542

[B17] Liu Y. C. Lin Y. C. Kanehara K. Nakamura Y. (2019). A methyltransferase trio essential for phosphatidylcholine biosynthesis and growth 1. *Plant Physiol.* 179 433–445. 10.1104/pp.18.01408 30518673 PMC6426410

[B18] Malito E. Sekulic N. Too W. C. S. Konrad M. Lavie A. (2006). Elucidation of Human Choline Kinase Crystal Structures in Complex with the Products ADP or Phosphocholine. *J. Mol. Biol.* 364 136–151. 10.1016/J.JMB.2006.08.084 17007874 PMC1885479

[B19] Marks M. D. Wenger J. P. Gilding E. Jilk R. Dixon R. A. (2009). Transcriptome Analysis of Arabidopsis Wild-Type and gl3–sst sim Trichomes Identifies Four Additional Genes Required for Trichome Development. *Mol. Plant* 2:803. 10.1093/MP/SSP037 19626137 PMC2713768

[B20] Meinke D. Muralla R. Sweeney C. Dickerman A. (2008). Identifying essential genes in Arabidopsis thaliana. *Trends Plant Sci.* 13 483–491. 10.1016/j.tplants.2008.06.003 18684657

[B21] Meinke D. W. (2020). Genome-wide identification of EMBRYO-DEFECTIVE (EMB) genes required for growth and development in Arabidopsis. *New Phytol.* 226 306–325. 10.1111/nph.16071 31334862

[B22] Muller H. J. (1932). Further studies on the nature and causes of gene mutations. *Int. Congr. Genet.* 61 213–255.

[B23] Nakamura Y. Andrés F. Kanehara K. Liu Y. C. Dörmann P. Coupland G. (2014). Arabidopsis florigen FT binds to diurnally oscillating phospholipids that accelerate flowering. *Nat. Commun.* 5:3553. 10.1038/ncomms4553 24698997 PMC3988816

[B24] Ruelland E. Valentova O. (2016). Editorial: lipid Signaling in Plant Development and Responses to Environmental Stresses. *Front. Plant Sci.* 7:324. 10.3389/FPLS.2016.00324 27014335 PMC4794493

[B25] Schneeberger K. Ossowski S. Lanz C. Juul T. Petersen A. H. Nielsen K. L. (2009). SHOREmap: simultaneous mapping and mutation identification by deep sequencing. *Nat. Methods* 6 550–551. 10.1038/nmeth0809-550 19644454

[B26] Schneider C. A. Rasband W. S. Eliceiri K. W. (2012). NIH Image to ImageJ: 25 years of image analysis. *Nat. Methods* 9 671–675. 10.1038/nmeth.2089 22930834 PMC5554542

[B27] Shuai B. Reynaga-Pe— na C. G. Springer P. S. (2002). The Lateral Organ Boundaries Gene Defines a Novel, Plant-Specific Gene Family. *Plant Physiol.* 129 747–761. 10.1104/PP.010926 12068116 PMC161698

[B28] Silverblatt-Buser E. W. Frick M. A. Rabeler C. Kaplinsky N. J. (2018). Genetic interactions between BOB1 and multiple 26S proteasome subunits suggest a role for proteostasis in regulating Arabidopsis development. *G3 Genes Genomes Genet.* 8 1379–1390. 10.1534/g3.118.300496 29487187 PMC5873925

[B29] Susila H. Jurić S. Liu L. Gawarecka K. Chung K. S. Jin S. (2021). Florigen sequestration in cellular membranes modulates temperature-responsive flowering. *Science* 373 1137–1142. 10.1126/SCIENCE.ABH4054 34516842

[B30] Tannert M. Balcke G. U. Tissier A. Köck M. (2021). At4g29530 is a phosphoethanolamine phosphatase homologous to PECP1 with a role in flowering time regulation. *Plant J.* 107 1072–1083. 10.1111/TPJ.15367 34098589

[B31] Tasseva G. Richard L. Zachowski A. (2004). Regulation of phosphatidylcholine biosynthesis under salt stress involves choline kinases in Arabidopsis thaliana. *FEBS Lett.* 566 115–120. 10.1016/j.febslet.2004.04.015 15147879

[B32] Tavasoli M. Lahire S. Reid T. Brodovsky M. McMaster C. R. (2020). Genetic diseases of the Kennedy pathways for membrane synthesis. *J. Biol. Chem.* 295 17877–17886. 10.1074/JBC.REV120.013529 33454021 PMC7762932

[B33] Waese J. Fan J. Pasha A. Yu H. Fucile G. Shi R. (2017). ePlant: visualizing and Exploring Multiple Levels of Data for Hypothesis Generation in Plant Biology. *Plant Cell* 29 1806–1821. 10.1105/TPC.17.00073 28808136 PMC5590499

[B34] Wang X. Chapman K. D. (2013). Lipid signaling in plants. *Front. Plant Sci.* 4:216. 10.3389/FPLS.2013.00216 23818891 PMC3694225

[B35] Welti R. Li W. Li M. Sang Y. Biesiada H. Zhou H. E. (2002). Profiling membrane lipids in plant stress responses: role of phospholipase Dα in freezing-induced lipid changes in arabidopsis. *J. Biol. Chem.* 277 31994–32002. 10.1074/jbc.M205375200 12077151

[B36] Wharfe J. Harwood J. L. (1979). Lipid metabolism in germinating seeds: purification of ethanolamine kinase from soya bean. *Biochim. Biophys. Acta Lipids Lipid Metab.* 575 102–111. 10.1016/0005-2760(79)90135-8 228735

[B37] Yunus I. S. Liu Y. C. Nakamura Y. (2016). The importance of SERINE DECARBOXYLASE1 (SDC1) and ethanolamine biosynthesis during embryogenesis of Arabidopsis thaliana. *Plant J.* 88 559–569. 10.1111/TPJ.13278 27436233

